# Supplementary material for: Management outcomes of secondary glaucoma due to retinopathy of prematurity: A 19-year prospective study at a tertiary eye care Institute. The Indian Twin cities ROP Screening (ITCROPS) database report number 8
Source: PLoS One. 2020 Sep 10;15(9):e0238633. doi: 10.1371/journal.pone.0238633 (PMC7482932; doi:10.1371/journal.pone.0238633)
Supplement: Attachment — Submitted filename: Response to Reviewers comments_PLOS one_ROP.docx [file pone.0238633.s001.docx]

**Response to Reviewers Comments**

**Reviewer #1:** The authors report the largest cohort to date of glaucoma secondary to ROP in a comprehensive and thorough fashion. Statistical analysis is performed in a sound way and results support this study's conclusions.

Response: We that the reviewer for the compliment and encouraging words.

They are invited to consider the following few comments to strengthen their work:

1. Out of 82 eyes included in this study, only 14 had VA assessment in the final follow-up. Please add mean follow-up for the whole cohort in terms of VA assessment. This would help the Editor and potential reading audience understand to what extent the lack of ‘final VA’ in the vast majority of this cohort weakens the secondary outcome of this study

Response: Thank you for the suggestion. We have provided the details of mean follow up for the visual acuity assessment in the abstract as well as in the results. Page 2, lines 41,42, page 7, lines 149, 150.

Mean follow up was 3.5±4.2 years in those whose Log MAR visual acuity was recorded. The mean follow up for the entire cohort was 1.14±2.24 years.

2. 34 of 54 eyes included in this study were stage V which explains the relatively high rates of secondary glaucoma and vitreoretinal surgery in this cohort compared to the literature.

Response: Yes, we agree that the incidence of glaucoma is high in eyes with stage V ROP that are untreated, however, it seems higher even post VR surgery.

Minor comments
3. Abstract lines 32-33 should read: Eighty-two eyes of 54 children with secondary glaucoma due to ROP where included in this study

Response: We have changed this as suggested. Page 2, line 33.

4. Line 60 should read: ‘These include’ instead of ‘they are’

Response: We have changed as suggested. Page 3, line 62.

5. Line 140 PCG should be explained when first used

Response: Sorry about this. We have expanded the abbreviation. Page 6, line 145.

**Reviewer #2:**

In the paper by Senthil et al, they report a large cohort of premature babies diagnosed with ROP, a subset of whom who were found to have concurrent glaucoma. There are two aspects to this paper – the first is the reporting of the prevalence of glaucoma and types of interventions in patients with ROP, and the second is estimating correlations between ophthalmic findings or interventions and stage of ROP in patients with glaucoma. The size of the cohort and the manner in which patients were collected is sound and robust, and though this is not the first paper to review glaucoma in ROP patients (Chandra et al., for example), it is the largest and verifies previous findings. It also reiterates that glaucoma prevalence goes up as ROP stage worsens, and one must not miss this diagnosis when evaluating patients with ROP. It is important to publish such data. I have concerns about the statistical approach for the second aspect of the paper, which is reporting correlations between ophthalmic findings or type of glaucoma and stage of ROP.

While I believe the authors have strong data with good information to report, the representation of the data and statistical approach is concerning and should be revised on multiple levels.

Response: As suggested by the reviewer, we have revised the data representation and statistical analysis.

1. I cannot replicate the chi-squared results. In Table 1, I assume this may be due to the way patients and eyes are treated, and so I will skip this table. I cannot replicate the exact p-values in Table 2. For example – Laser indirect ophthalmoscopy, category “0”. If I ignore the first column as the authors say they did (due to sample size), I get a p-value of 0.0002 for a 2x3 chi-squared contingency table where the rows are “0” and “not 0” and the columns are stage of ROP. The authors report a p-value of 0.50. I am having this same problem for some – if not all – of the data. The authors need to clarify significantly exactly what test they are doing to yield these results and what is the denominator. But there are further concerns about the approach as outlined below. Of note, I can replicate the subsequent post-hoc pairwise chi-squared tests that were done to identify the source of a signal.

Response: The tables were modified and should reflect the correct values now. Tables 1-4, and the statistical analysis.

2. For the continuous variables, Kruskal-Wallis (and subsequently individual Mann-Whitneys) are used, and this is appropriate.

Response: We thank the reviewer for the comment.

3. For the other variables that have been treated as categorical, it gets a bit more nuanced. In general, it is misleading to calculate a separate chi-square for each subcategory within a category (for example – treating number of lasers 0,1,2 as three categories and calculating separate statistics). Within a category, these tests are not independent – if an eye takes on one subcategory, it is by definition not the other. Rather, one can do a multi-way chi-squared test on the full 3x3 (or 2x3 or 4x3, depending on the category) contingency table, and then report the pairwise comparisons therein that likely represent the signal, as the authors have done in the Table caption. This results in one p-value per category.

Response: We understand the concern. We have simplified to reflect them better. Analysis were done for 2x3 tables now resulting in one p-value per category.

4. A Fisher’s test should have been used rather than a chi-squared if any category has 10 or fewer total counts.

Response: As suggested by the reviewer, we have used Fisher Exact test for multiple pair-wise comparisons now.

5. It is sub-optimal to use the chi-squared test on ordinal categorical data. I would suggest a Kruskal-Wallis for these data, but there are other options the authors could choose as well. For example – the number of lasers (0,1,2) is converted into 3 independent categories; these are ordered and not unrelated, so they should be treated as such.

Response: The tables were modified and the ordinal categorical data were eliminated in order to avoid confusion.

6. The Bonferonni correction is unusual. The subsequent pairs of mann-whitneys (or chi-squares) after the multi-way Kruskal-wallis (or chis-square) are not at all independent from each other, and therefore it doesn’t make sense to correct with Bonferonni (and correcting for 2 tests when more than 2 were done is also incorrect). If the authors want to correct for multiple testing, consider a Dunn’s post-hoc correction in this setting after a Kruskal-Wallis (or consult with their biostatistician on the best way to do this). In addition, the real Bonferonni correction that should have been done is to correct for the number of categories that were tested, as these are all independent of one another (for example, in Table 2, correct for 5 tests -- # of VR surgeries, age, laser, avastin, lensectomy).

Response: After consulting with our bio-statistician, we have represented the data in a simpler way. The tables were revised and appropriate tests were applied now considering the previous comments of Reviewer #2.

These problems make it somewhat hard and confusing to read the paper and glean the important takeaways.

Response: We have worked towards eliminating these problems during revision in order to avoid confusion reading the paper.

Finally, one other question for the authors is why they are calculating statistics within the glaucoma group and not comparing glaucoma to non-glaucoma? For example – the number of Avastin injections in patients who developed or didn’t develop glaucoma? They have a wealth of data and these seem like the important questions.

Response: We thank the reviewer for the suggestion. We agree with the suggestion, however it is beyond the scope of this manuscript.

I do believe that overall, the trend of their results will be similar with the above corrections, but because of these concerns, it was hard to dive deeper into interpreting the results. They should consult with someone who has training in statistics in order to clean up the results and form robust conclusions.

Response: We have re-evaluated the results and made necessary corrections. We agree that the results did not change however there was a small variation in the numbers. Hope the analysis and results are fine now.
